# BITS 2017: the annual meeting of the Italian Society of Bioinformatics

**DOI:** 10.1186/s12859-018-2295-y

**Published:** 2018-10-15

**Authors:** Giuliano Armano, Giorgio Fotia, Andrea Manconi

**Affiliations:** 1 Dept. of Electrical and Electronic Engineer, Univ. of Cagliari, P.zza D’Armi, Cagliari, 09123 Italy; 2 Center for Advanced Studies, Research and Development in Sardinia, Loc. Pixina Manna, Cagliari, 09010 Pula Italy; 3 National Research Council, Institute for Biomedical Technologies, Via F.lli Cervi, 93, Segrate, 20090 MI Italy

**Keywords:** BITS, Bioinformatics, Meeting of the Italian Society of Bioinformatics

## Abstract

This preface introduces the content of the BioMed Central journal Supplement related to the 14th annual meeting of the Bioinformatics Italian Society, held in Cagliari, Italy, from the 5th to the 7th of July, 2017.

## BITS, the Italian Society of Bioinformatics

BITS, the Italian Society of Bioinformatics [1], is the largest non-profit association of researchers involved in Bioinformatics with work activities or interest in Italy. The primary aim of BITS is to join the research scientists interested in Bioinformatics, meant as a multi-disciplinary science for the study of biological systems at the molecular and cellular level by using informatics and computational methods and models. Main goals of the association are the study, development and spreading of Bioinformatics in the scientific, academic, technological and industrial environment. Since its foundation, BITS has continuously increased the number of members and was recognized as a Regional group of the International Society for Computational Biology (ISCB). BITS promotes activities as courses and workshops, at national and international level. Such events were mainly located in Italy and, in some cases, abroad.

## BITS 2017 annual meeting

The fourteenth annual meeting of BITS has been held in Cagliari, from the 5th to the 7th of July, 2017. The meeting was organized by Giuliano Armano, Andrea Manconi, Alessandro Orro, Giorgio Fotia and Francesco Cucca, together with a Scientific Committee including most of the Italian Bioinformatics senior scientists (see Table 1). About 100 participants attended the meeting. The scientific program included the following keynote speakers:

**Table 1:** BITS 2017 Program committee

Claudia Angelini	CNR-IAC, Napoli, Italy
Roberta Bosotti	Nerviano Medical Sciences, Nerviano (MI), Italy
Raffaele Calogero	University of Torino, Italy
Rita Casadio	University of Bologna, Italy
Michele Caselle	University of Torino, Italy
Arnaud Ceol	IIT, Milano, Italy
Federica Chiappori	CNR-ITB, Milano, Italy
Domenica D’Elia	CNR-ITB, Bari, Italy
Angelo Facchiano	CNR-ISA, Avellino, Italy
Manuela Helmer-Citterich	University of Rome “Tor Vergata”, Italy
Vito Flavio Liciulli	CNR-ITB, Bari, Italy
Giosuè Lo Bosco	University of Palermo, Italy
Paolo Magni	University of Pavia, Italy
Anna Marabotti	University of Salerno, Italy
Roberto Marangoni	University of Pisa, Italy
Marco Masseroli	Politecnico di Milano, Italy
Giancarlo Mauri	University of Milano-Bicocca, Milan
Luciano Milanesi	CNR-ITB, Milano, Italy
Ettore Mosca	CNR-ITB, Milano, Italy
Alessandro Pandini	Brunel University London, UK
Marco Pellegrini	CNR-IIT, Italy
Enresto Picardi	University of Bari, Italy
Alfredo Pulvirenti	University of Catania, Italy
Paolo Romano	IRCCS San Martino IST, Genova, Italy
Remo Sanges	SZN, Naples, Italy
Roberto Tagliaferri	University of Salerno, Italy
Silvio Tosatto	University of Padua, Italy
Luigi Varesio	Giannina Gaslini Institute, Genoa, Italy
Andreas Zanzoni	Aix-Marseille Université, UMR1090 TAGC, France

- Prof. Manuela Helmer-Citterich (University of Rome Tor Vergata, Italy)
- Prof. Dominik Heider (University of Marburg, Germany)
- Dr. Alexander Kel (GeneXplain, Germany)
- Prof. Pietro Liò (University of Cambridge, UK)
- Prof. Andrew C.R. Martin (University College London, UK)
- Dr. Paolo Missier (Newcastle University, UK)

Participants were invited to submit scientific contributions, as oral presentations or posters. After evaluation of the 82 abstracts received, the Scientific Committee selected 28 of them for oral presentations, and accepted 54 as posters. The Conference program was organized into the following sessions: *Disease Genomics and Next Generation Sequencing*; *Protein structure and function*; *Systems Biology; Algorithms for Bioinformatics*; *Databases and Big Data application in Bioinformatics*;*Molecular Evolution; Genome 3D: Bioinformatics, computing infrastructure and opportunities for Chromatin Conformation Analysis*.

## BITS 2017 supplement to BMC Bioinformatics journal

After the meeting, all authors of scientific contributions have been invited to prepare and submit a manuscript to be evaluated for publication in a BioMed Central Bioinformatics journal Supplement. Manuscripts have been peer-reviewed in agreement with BMC rules for supplements’ manuscripts evaluation. At the end of this process, 8 articles have been accepted and included in this supplement. A short presentation of each contribution is reported in the following section.

## BMC Bioinformatics supplement content

The articles accepted for publication in the BMC Bioinformatics supplement devoted to BITS 2017 cover different topics, including novel algorithms, applications, comparisons of methods for analysing specific data, and tool developments. A brief summary of the accepted articles follows hereinafter.

- Kulkarni et al. [2] – *Reproducible Bioinformatics Project: A community for reproducible bioinformatics analysis pipelines*.
  This paper illustrates the Reproducible Bioinformatics Project (RBP), a non-profit and open-source project, whose aim is to provide a schema and an infrastructure, based on docker images and R package, to provide reproducible results in Bioinformatics. RBP provides a general schema and an infrastructure to distribute robust and reproducible workflows, guaranteeing to final users the ability to repeat consistently any analysis independently by the used UNIX-like architecture.
- Bonnici et al. [3] – *cuRnet: an R package for graph traversing on GPU*.
  This paper illustrates cuRnet, an R package for graph traversing. The package provides a GPU-based implementation of the following algorithms: i) breath-first search, ii) single-source shortest paths, and iii) strongly connected components. The authors performed experiments aimed at testing cuRnet on a benchmark of large protein interaction networks and at interpreting high-throughput omics data through network analysis. Performances, in terms of execution time, are compared with those of the corresponding sequential implementations provided in the iGraph R package.
- Bonnici et al. [4] – *Arena-Idb: a platform to build human non-coding RNA interactions networks*.
  In this paper, the authors describe a platform called Arena-Idb, aimed at retrieving comprehensive and non-redundant annotated ncRNAs interactions. The platform provides a framework for reconstructing the network of ncRNAs heterogeneous interactions (i.e., with other types of molecules) and their relationships with human diseases. The latter aspect guides the integration of data, which are extracted from different sources, via mapping of entities and minimization of ambiguity. Arena-Idb provides a schema and a visualization system to integrate ncRNA interactions that assists in discovering ncRNA functions through the extraction of heterogeneous interaction networks.
- Weitschek et al. [5] – *CamurWeb: A classification software and a large knowledge base for gene expression data of cancer*.
  This paper introduces *CamerWeb*, a web-based tool to extract multiple rule-based classification models from RNA sequencing experiments and to create a large knowledge base of these rules. Authors performed experiments to prove the validity of CamurWeb, obtaining many classification models and thus several genes that are associated to 21 different cancer types. All extracted knowledge, classification results, and selected genes were made public on the CamurWeb platform.
- Moscatelli et al. [6] – *An infrastructure for Precision Medicine through analysis of Big Data*.
  In this work, the authors present a new information technology infrastructure able to efficiently integrate large volumes of heterogeneous biological data. The proposed infrastructure was devised for the Italian Diagnostic Center (CDI), a large Italian medical center. Results on a large set of data show that complex questions that can be used in a variety of fields (such as predictive and precision medicine) can be answered through the integration of data aggregation, data classification and appropriate statistical methods.
- Petrini et al. [7] – *A GPU-based algorithm for fast node label learning in large and unbalanced biomolecular networks*.
  In this paper the authors propose a novel semi-supervised GPU-based parallelization of COSNet, an imbalance-aware algorithm build on a Hopfield neural model recently proposed for solving the automated protein function prediction problem. The authors demonstrated that by parallelizing COSNet they achieved on average a speed-up of 180x in solving the cited problem for the S. cerevisiae, Mus musculus and Homo sapiens organisms, while lowering memory requirements.
- Casiraghi et al. [8] – *A novel computational method for automatic segmentation, quantification and comparative analysis of immunohistochemically labeled tissue sections*.
  In this paper the authors describe MIAQuant_Learn, a software that segments, quantifies and analyzes markers in histochemical and immunohistochemical images of different provenance. Applications of MIAQuant_Learn in clinical research studies prove its effectiveness as a reproducibile, fast and efficient tool for automatic extraction, quantification and analysis of histological sections. Its robustness with respect to several deficits caused by image acquisition systems has also been highlighted.
- Merlotti et al. [9] – *Statistical modelling of CG interdistance across multiple organisms*
  Previous analyses on CG dinucleotide position along the genome allowed to highlight its epigenetic role in DNA methylation. In this paper this analysis is extended over a selected set of higher-order organisms. The best fitting probability density function is then applied to a large range of organisms, to let emerge some relevant global features. The corresponding research finding, obtained in a comparative setting, is that the Gamma distribution is optimal. The quantification of statistical properties of CG dinucleotide positioning along the genome is confirmed as a useful tool to characterize broad classes of organisms, spanning over the whole range of biological complexity.
  *Giuliano Armano*, DIEE, Univ. of Cagliari, Italy
  *Giorgio Fotia*, CRS4, Pula (Cagliari), Italy
  *Andrea Manconi*, CNR-ITB, Segrate (Milano), Italy
